# Workarounds Emerging From Electronic Health Record System Usage: Consequences for Patient Safety, Effectiveness of Care, and Efficiency of Care

**DOI:** 10.2196/humanfactors.7978

**Published:** 2017-10-05

**Authors:** Vincent Blijleven, Kitty Koelemeijer, Marijntje Wetzels, Monique Jaspers

**Affiliations:** ^1^ Center for Marketing & Supply Chain Management Nyenrode Business University Breukelen Netherlands; ^2^ Department of Medical Informatics Academisch Medisch Centrum University of Amsterdam Amsterdam Netherlands; ^3^ Emma Children's Hospital Academisch Medisch Centrum University of Amsterdam Amsterdam Netherlands

**Keywords:** electronic health records, qualitative research, physicians, nurses, patient safety, quality of health care, efficiency, workflow

## Abstract

**Background:**

Health care providers resort to informal temporary practices known as workarounds for handling exceptions to normal workflow unintendedly imposed by electronic health record systems (EHRs). Although workarounds may seem favorable at first sight, they are generally suboptimal and may jeopardize patient safety, effectiveness of care, and efficiency of care.

**Objective:**

Research into the scope and impact of EHR workarounds on patient care processes is scarce. This paper provides insight into the effects of EHR workarounds on organizational workflows and outcomes of care services by identifying EHR workarounds and determining their rationales, scope, and impact on health care providers’ workflows, patient safety, effectiveness of care, and efficiency of care. Knowing the rationale of a workaround provides valuable clues about the source of origin of each workaround and how each workaround could most effectively be resolved. Knowing the scope and impact a workaround has on EHR-related safety, effectiveness, and efficiency provides insight into how to address related concerns.

**Methods:**

Direct observations and follow-up semistructured interviews with 31 physicians, 13 nurses, and 3 clerks and qualitative bottom-up coding techniques was used to identify, analyze, and classify EHR workarounds. The research was conducted within 3 specialties and settings at a large university hospital. Rationales were associated with work system components (persons, technology and tools, tasks, organization, and physical environment) of the Systems Engineering Initiative for Patient Safety (SEIPS) framework to reveal their source of origin as well as to determine the scope and the impact of each EHR workaround from a structure-process-outcome perspective.

**Results:**

A total of 15 rationales for EHR workarounds were identified of which 5 were associated with persons, 4 with technology and tools, 4 with the organization, and 2 with the tasks. Three of these 15 rationales for EHR workarounds have not been identified in prior research: data migration policy, enforced data entry, and task interference.

**Conclusions:**

EHR workaround rationales associated with different SEIPS work system components demand a different approach to be resolved. Persons-related workarounds may most effectively be resolved through personal training, organization-related workarounds through reviewing organizational policy and regulations, tasks-related workarounds through process redesign, and technology- and tools-related workarounds through EHR redesign efforts. Furthermore, insights gained from knowing a workaround’s degree of influence as well as impact on patient safety, effectiveness of care, and efficiency of care can inform design and redesign of EHRs to further align EHR design with work contexts, subsequently leading to better organization and (safe) provision of care. In doing so, a research team in collaboration with all stakeholders could use the SEIPS framework to reflect on the current and potential future configurations of the work system to prevent unfavorable workarounds from occurring and how a redesign of the EHR would impact interactions between the work system components.

## Introduction

### Growing Adoption of Electronic Health Record Systems

Electronic health record systems (EHRs) can improve the way medical information is stored, communicated, and processed by those involved in delivering health care [1]. Adopting EHRs may result in favorable outcomes related to patient safety [2-4], quality of care [4-6], efficiency [4,7-10], and reduced costs [11,12]. In pursuit of these benefits and support provided by governmental stimuli programs [13], an increasing number of hospitals around the world have adopted EHRs [14-16].

Although adoption rates of EHRs are rising [17], realizing the promising benefits of adopting EHRs is far from evident. Many studies address unfavorable and often unanticipated outcomes of adopting EHRs. Examples include health care providers suffering from poor navigation [18,19], difficulties in finding the right information in the EHR [20], not all clinical work being supported by EHRs [21], never ending system demands [22], and significantly disrupted workflows due to modified timing, sequence of work practices, and revised professional responsibilities [23-26].

### Workarounds to Electronic Health Record System Usage

Many causes of unfavorable outcomes of adopting EHRs can be traced back to discrepancies between the behavior and intentions of EHR users and the workflow as dictated by the EHR—often termed workflow mismatches [22,23,27-29]. Health care providers develop workarounds when they perceive EHR usage negatively affecting their practices as a consequence of workflow mismatches [21,30,31]. Workarounds are defined as “informal temporary practices for handling exceptions to normal workflow [32]” that “do not follow explicit or implicit rules, assumptions, workflow regulations, or intentions of systems designers [33].” Workarounds allow EHR users to proceed in accomplishing their tasks, in particular when under conditions of high time pressure [32]. Identified reasons for EHR workarounds include a perceived lack of efficiency, task complexity dictating workflow issues, no desired option being available in the system-dictated workflow, and a lack of trust in electronic versus paper-based communication [21,32,34-37].

Workarounds are double-edged swords. They may improve workflow efficiency [30,38], situationally increase patient safety by, for example, overriding alerts to get critical medication to a patient as soon as possible [27], or assist physicians when they purposefully order a wrong drug to trigger the alert system to suggest the right one [27]. However, workarounds frequently also lead to unstable, unavailable, or unreliable information or work protocols [32]. They may negatively influence the safety, effectiveness of care, and efficiency of care. For example, workarounds may bypass important security blocks (eg, working in a so-called emergency mode in nonemergency situations and thereby omitting security checks) [33] or lead to administering medication to the wrong patient or in incorrect doses [31,33]. Furthermore, they cloak deficiencies as devising workarounds rather than bringing problems to the attention of systems designers causes problems to remain hidden, which simultaneously inhibits optimization [39]. Finally, workarounds undermine standardization by using an alternative way to accomplish a task, thereby not conforming to a system-enforced way of working designed to safeguard patient safety or to eliminate variability [40,41]. Given their potentially adverse influence, research into workarounds has a prominent place in health care, and workarounds have been identified, analyzed, and described for various systems (eg, medication delivery systems, electronic medical records, and barcode medication administration systems), in various contexts (eg, academic vs nonacademic hospitals), and ways (eg, direct observations, expert panels, and surveys) [36,42-47].

### Contribution

Existing literature primarily provides insight into types of EHR workarounds (paper and computer-based) [21,36], multiple reasons behind EHR workaround creation such as data confidentiality concerns, system instability, resistance to change, task complexity, knowledge gaps, and a perceived lack of efficiency [21,36,48-51], and describes several key features of EHR workarounds such as workarounds being avoidable or unavoidable, deliberately chosen or unplanned, and temporary or routinized [47]. However, research into the scope and impact of EHR workarounds on patient care processes is not as extensive. Knowledge of the scope and impact of EHR workarounds on organizational workflows and outcomes of care services is limited, and there are 2 areas of concern. First, concerning the scope of EHR workarounds, knowing whether a workaround solely affects the single EHR user who devised it or whether its effect extends beyond the EHR user to the work context of other health care providers is key to infer its impact on the overall patient care workflow. Second, knowing whether the impact of an EHR workaround is favorable or unfavorable, for example the influence it has on EHR-related safe and effective and efficient delivery of care, provides insight into how to address these concerns. This study addresses these issues and contributes to existing literature in 2 ways. On the basis of a large case study, we present an overview of 15 bottom-up identified rationales for EHR workarounds and give a definition for each rationale. In addition, for the most prominent workarounds identified per rationale, their scope and impact on patient safety, effectiveness of care, and efficiency of care are analyzed and discussed from a sociotechnical perspective using the Systems Engineering Initiative for Patient Safety (SEIPS) framework [52]. The research question central to this study was as follows: “What EHR workarounds are developed by health care professionals during their ordinary medical practice, and what are their rationales, scope, and impact on patient safety, effectiveness of care, and efficiency of care?”

## Methods

### Study Design

To identify and analyze EHR workarounds, a case study was performed at one of the largest university hospitals in the Netherlands. The hospital adopted a hospital-wide EHR in 2015. Over 8000 hospital staff work with the EHR, and 100% of all orders (eg, medication, blood tests, lab results, and x-rays) are entered through the EHR. Enforced by strict hospital policies, paper-based orders are no longer accepted. The EHR, purchased from a large US EHR vendor, is an integrated suite of health care software. Its applications support functions related to patient care and management, registration and scheduling, clinical systems for health care providers, ancillary laboratory, pharmacy and radiology systems, and a billing system.

**Figure 1:**
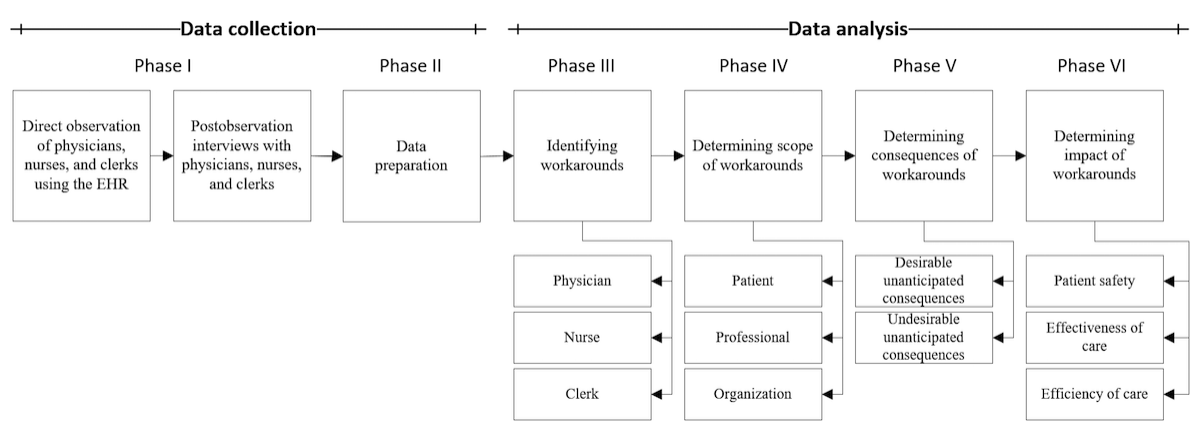
Illustration of the 6 research phases.

The research project involved 6 major chronological phases, as illustrated in Figure 1. The following subsections address the data collection phases (I and II) and data analysis phases (III, IV, V, and VI) in greater detail. A summary of the data collection and analysis setup used for all 3 settings is provided in Table 1.

A more comprehensive description of the research approach taken for this study has been published as a study protocol [53].

### Data Collection

We adopted a qualitative approach consisting of nonparticipant direct observation combined with semistructured follow-up interviews with physicians, nurses, and clerks using the EHR while performing their ordinary medical practice. The observations allowed us to observe workarounds while work practices and EHR use by health care professionals unfold in situ [54]. The semistructured follow-up interviews allowed us to gain greater insight into each observed workaround, more specifically their scope (ie, patient, professional, and organization) and impact (ie, consequences for patient safety, effectiveness of care, and efficiency of care).

**Table 1:** Summary of research design by process studied.

Process	Preparing outpatient consultation	Providing outpatient consultation	Providing inpatient consultation
Sample	14 physicians and 5 nurses (same staff as in providing outpatient consultation process)	14 physicians, 5 nurses, and 3 clerks (same staff as in preparing outpatient consultation process)	17 physicians and 8 nurses (nurses perform clerical tasks)
Participant selection criteria	Must have completed the required training to use EHR	Must have completed the required training to use EHR	Must have completed the required training to use EHR
	Must have used EHR from the moment of its implementation	Must have used EHR from the moment of its implementation	Must have used EHR from the moment of its implementation
Setting	Private office	Examination room	Inpatient ward
Interaction	User-system	User-patient and user-system	User-patient and user-system
Procedure (per person)	Direct observation while preparing outpatient consultation, asking opportunistic questions while observing, and semistructured follow-up interviews	Direct observation while providing outpatient consultation and semistructured follow-up interviews	Direct observation during ward rounds and postward round EHR usage and semistructured follow-up interviews
Data analysis	Transcribing and subsequent bottom-up coding of audiovisual recordings in ATLAS.ti.	Transcribing and subsequent bottom-up coding of audiovisual recordings in ATLAS.ti.	Transcribing and subsequent bottom-up coding of audiovisual recordings in ATLAS.ti.

A total of 31 physicians, 13 nurses, and 3 clerks were observed and interviewed (see Table 1). These numbers were not fixed beforehand: observations and interviews continued till the research team agreed that data saturation was achieved. Participants were recruited via the director of medical staff and director of operations, as well as participants suggesting other participants. Data were gathered within 3 clinical specialties: pediatrics, gynecology, and internal medicine. All these specialties use the same EHR of which the look and feel is identical, although additional specific functionalities tailored to each specialty are used. Within each specialty, providers were observed while using the EHR in 3 distinct processes: the preparation of outpatient consultation, providing outpatient consultation, and providing inpatient consultation. All direct observations and interviews were audiovisually captured by a small and unobtrusive camera positioned at a static location facing the monitor displaying the EHR. All physicians, nurses, clerks, and patients were asked for an informed consent before any recording took place. In total, around 200 hours of audiovisual material was captured.

The recordings were transcribed by VB in separate Microsoft Word documents and imported into a software application named ATLAS.ti. Within these imported documents, quotations were created for selected text sections or video frames possibly relating to an EHR workaround. After processing all transcriptions, VB reviewed each transcription, followed by KK and MJ validating the transcriptions and quotations to ensure (1) quotations indeed related to a workaround, (2) there was consistency among the quotations in terms of the range of the selected data, (3) minimal discrepancies existed between the audiovisual data and transcribed text, and (4) no relevant sections of data were overlooked.

### Data Analysis

A bottom-up (ie, inductive) approach to coding was followed [55]. A provisional coding taxonomy containing multiple rationales for EHR workarounds was first created based on impressions and notes taken during each observation and interview. Before actual coding started, the coding team consisting of 2 independent coders (VB and an external [communications] researcher) was instructed on the EHR, the coding taxonomy, the meaning of each code, and the basics of coding in ATLAS.ti. To safeguard coding quality, the coding team coded the same copy of several random interview transcriptions using the provisional coding taxonomy. Copies of both coders were compared and discrepancies and ambiguities were discussed and resolved.

After the provisional coding taxonomy was finalized, the coding team began open coding. New codes or alternative code names could be proposed when data did not fit into the provisional taxonomy codes. Discrepancies in terms of codes assigned to the same quotation were resolved through discussion with the coders and MJ. The provisional coding taxonomy was adjusted when necessary. The tentative coding taxonomy developed itself over time into a final set of codes. The majority of the transcriptions was independently coded. Moreover, 25% of the transcriptions were coded by both coders. For these transcriptions, inter-rater reliability was calculated to be 0.72 and inter-rater agreement was 0.93.

After coding all transcriptions, VB, KK, MW, and MJ analyzed each workaround in terms of its source of origin, scope, and impact. To facilitate this, we adapted one of the most widely used health care human factors systems frameworks, the SEIPS framework [52] (see [53] for more details). With the integrated and holistic perspective of the SEIPS framework, relationships between a health care work system (including workarounds), processes, and outcomes can be studied. The SEIPS framework has already proven valuable in studying workarounds in various health care contexts [33,56]. The adapted SEIPS framework is explained in greater detail in the Results section.

The first data analysis step after coding concerned determining the scope of each workaround by studying which stakeholders (ie, patient, professional, organization, or a combination thereof) were actually affected by the workaround. In the second step, the consequences of each workaround were determined, and each consequence was labeled as desirable or undesirable [27]. In the final step, the impact of each workaround consequence on the safe, effective, and efficient delivery of care was determined. The impact of each workaround was determined by analyzing the audiovisual fragments and related transcriptions of the direct observations and, in particular, the follow-up interviews conducted with the observed health care professionals using the workarounds. For most workarounds, their impact on patient safety, effectiveness of care, and efficiency of care was relatively clear: the impact was either directly visible or elaborated upon by the observed health care professionals at the moment of observing or clarified during the follow-up interviews. In case the impact could not be directly or accurately determined after the interviews, assistance from multiple other experts such as EHR developers, quality assurance staff, or hospital management was requested to provide additional insight. We define patient safety, effectiveness of care, and efficiency of care as follows:

- Patient safety is defined by the Institute of Medicine as “the prevention of harm to patients” [57]. In our context, we interpret this as any EHR-related incident resulting from a workaround which could possibly harm patients receiving care.
- The Institute of Medicine defines effective care as “providing services based on scientific knowledge to all who could benefit and refraining from providing services to those not likely to benefit, avoiding underuse and overuse, respectively” [58]. Workarounds may result in unstable, unavailable, or unreliable information on patient care (processes) or work protocols [32]. In our context, we interpret this as whether the workaround impacts the accuracy and completeness with which not just the single EHR user who created the workaround but the overall hospital staff in the case study hospital deliver care to patients (that is of proven value and has no significant tradeoff).
- The Institute of Medicine defines efficiency as “avoiding waste, including waste of equipment, supplies, ideas, and energy” [58]. We also interpret this as resources such as time or finances expended in relation to the accuracy and completeness with which EHR users achieve goals [59]. Research has shown that the ratio between provider-EHR system and provider-patient interaction demands careful balancing [60-62] and that EHRs claim a significant portion of physicians’ time and draw attention away from their direct interactions with patients and from their personal lives [63,64]. Workarounds may increase or decrease the efficiency through which EHR users achieve their goals.

## Results

### Rationales for Electronic Health Record System Workarounds

A total of 15 rationales for EHR workarounds could be identified from the audiovisual data. Table 2 provides an overview of the EHR workaround rationales including definitions.

**Table 2:** Identified rationales for EHR workarounds and their definitions.

Rationale for EHR workaround	Definition
Declarative knowledge	Not knowing how to use (a part of) the EHR to accomplish a task
Procedural knowledge	Knowing how but not being proficient enough to use a part of the EHR to accomplish a task
Memory aid	Writing patient data down on paper (eg, keywords) or adding visual elements to parts of text in a progress note (eg, boldfacing, italicizing, or underlining) to remind oneself
Awareness	Storing patient data that are perceived important by the EHR user for other colleagues to be noticed in a data field other than the intended field in the EHR
Social norms	Informal understandings among health care professionals leading to the creation and dissemination of workarounds (eg, mimicking workarounds devised by colleagues to accomplish a task or working around the system as friendly requested or enforced by a fellow clinician)
Usability	High behavioral user cost in accomplishing a task
Technical issues	(A part of the) EHR halting, crashing, or slowing down, hindering the EHR user in accomplishing a task
Data presentation	Preferring a different data view (eg, visualization by means of charts or graphs rather than plain text)
Patient data specificity	Needing to enter or request patient data with greater or lesser specificity than offered or enforced by the EHR
Task interference	Inability to perform multiple tasks at once (eg, simultaneously treating a patient on a treatment table as well as entering patient data into the EHR)
Commitment to patient interaction	Valuing patient interaction over computer interaction (ie, writing things down on paper and afterwards entering this into the EHR)
Efficiency	Using an alternative way to accomplish a task that improves actual efficiency
Data migration policy	Not having (direct) access to required historical data due to data not having been imported from previously used systems to the current EHR
Enforced data entry	EHR enforcing user to enter patient data of which neither the user nor the patient has knowledge of
Required data entry option missing	EHR not offering the required data entry option (eg, 3.75 mg prednisone rather than the available options of 2.5 mg or 5 mg)

**Figure 2:**
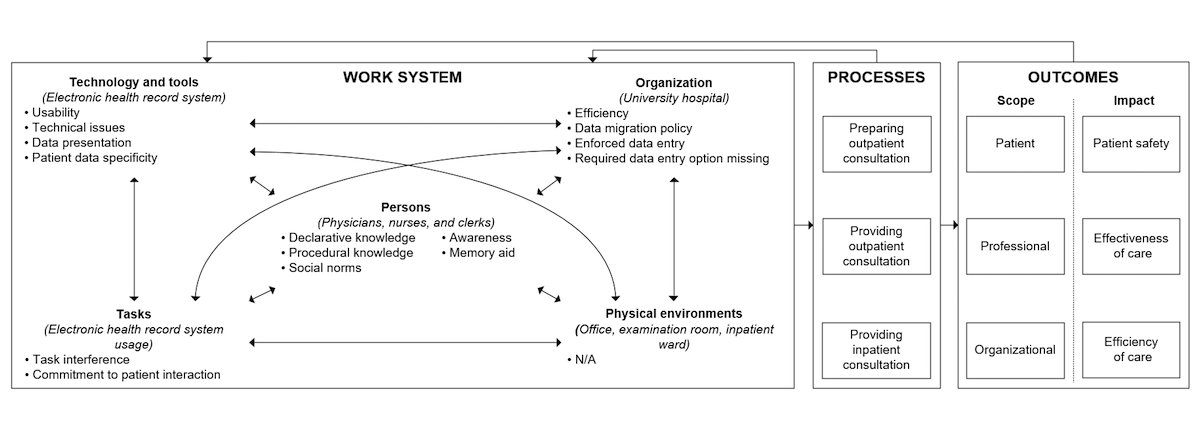
Conceptual framework used to study electronic health record system (HER) workarounds based on the Systems Engineering Initiative for Patient Safety (SEIPS) framework including an overview of the 15 identified rationales for EHR workarounds and the work system components they are associated with.

To analyze and determine the scope and impact of each EHR workaround, we used an adapted version of the SEIPS framework [52] tailored to our context (as discussed in Results). As illustrated in Figure 2, the framework consists of 3 main blocks that in turn consist of multiple components:

- The *work system* in which EHR workarounds are created, consisting of the observed and interviewed persons (physicians, nurses, and clerks), tools and technologies used (the EHR and other information systems), tasks performed (treating a patient on a treatment table or entering a patient’s medical history, ordering medication, etc in the EHR), case study organization (the university hospital), and the physical environments in which the case study participants were observed and interviewed (private offices, examination rooms, and inpatient wards). The arrows between the components illustrate their interrelated nature. The components may act simultaneously and jointly exert influence over processes and resulting outcomes.
- The 3 *processes* in which case study participants were observed and interviewed and in which the EHR workarounds were revealed: the preparation of outpatient consultation, providing actual outpatient consultation, and providing actual inpatient consultation.
- The *outcomes* of EHR workarounds in terms of their scope (patient, professional, organizational, or a combination thereof) and impact (patient safety, effectiveness of care, and efficiency of care).

To reveal their source of origin, each of the 15 identified rationales for EHR workarounds was associated with 1 of the 5 work system components (as shown in Figure 2). The following section discusses each component of the work system and their associated workaround rationales in greater detail.

### Electronic Health Record System Workarounds: Scope and Impact

The following subsections elaborate upon each component of the work system shown in Figure 2 and their associated workaround rationales. Per work system component, a table lists the associated rationales for EHR workarounds including several observed workarounds per rationale. For each workaround, its scope and potential impact on the safe, effective, and efficient delivery of care is listed.

The scope column indicates the degree of influence of a workaround, specifically which stakeholder(s) (may) experience (mostly unfavorable) consequences of the workaround. Within this column, P stands for patient, C for health care professional (clinician or clerk), O for the overall organization (eg, other specialties or hospital management), and a combination of these 3 letters for a combination of the foregoing stakeholders. Within the safety, effectiveness, and efficiency columns, ↑ denotes an increase, ↓ denotes a decrease, • denotes a negligible influence, and ? denotes undecided in case the impact of the workaround on patient safety, effectiveness of care, or efficiency of care could not be accurately determined (eg, impact being highly situational).

The amount of observed EHR workarounds is too large for all to be listed and discussed. We therefore highlight the most prominent workarounds and their consequences per workaround rationale and discuss their scope and potential impact.

#### Persons

The Persons component refers to the observed and interviewed physicians, nurses, and clerks [52]. The 5 rationales for workarounds in this category primarily concern human-related factors: declarative knowledge, procedural knowledge, memory aid, awareness, and social norms. Table 3 provides an overview of prominent Persons-related workarounds and their scope and potential impact.

##### Declarative Knowledge

Declarative knowledge-related workarounds resulted from EHR users not knowing how to accomplish certain tasks at hand in the EHR. Whenever this occurred, users argued that they either had not (yet) taken part in necessary training to accomplish a given task or that they did take part in training but considered it too superficial and as a result still had no knowledge of how to use (a part of) the EHR. For example, a physician did not know how to use the functionality that automatically imports relevant patient data from the EHR into a letter to be sent out to, for example, a general practitioner or fellow clinician. Instead, the physician manually reentered patient data from the EHR into a letter. The physician managed to proceed with her workflow, albeit in a less efficient way and the reentering of patient data being prone to mistakes. Another physician did not know how to sign the patient treatment plans she created. Although the EHR allowed her to proceed with her workflow without signing any treatment plans, other clinicians may be led to believe these treatment plans are still pending to be reviewed and signed, and as a consequence, patients may not receive proper care.

##### Procedural Knowledge

Procedural knowledge-related workarounds resulted from EHR users considering themselves insufficiently proficient (despite having taken part in training) to safely and correctly use a part of the EHR to accomplish certain tasks at hand. For this reason, users devised other ways which they were more comfortable with—at least for the time being—to accomplish their task. For example, during a patient consultation session, a physician wanted to order an allergy test so the patient could immediately make an appointment at the reception desk after the consultation session. However, the physician was unsure whether her draft order was filled out correctly. She requested a colleague to review her draft order later that day, which improved patient safety, but she had to send the patient home with the request to call for making an appointment the following day.

**Table 3:** Prominent EHR workarounds concerning Persons and their scope and potential impact (↑ denotes an increase, ↓ denotes a decrease, • denotes a negligible influence, and ? denotes undecided. P stands for patient, C for health care professional [clinician or clerk], and O for the overall organization).

Rationale	Workaround	Scope	Safety	Effectiveness	Efficiency
Declarative knowledge	Manually reentering patient data from the EHR into a letter due to not knowing how to use the automatic letter generation tool	C	↓	•	↓
	Asking colleagues for assistance when not knowing the correct referral codes when referring patients to colleagues of another specialty	C	•	•	↓
	Not registering treatments due to not knowing what treatments are supposed to be registered and which ones should not	O	•	↓	↑
	Not signing treatment plans due to not knowing how to	PO	↓	↓	•
	Asking colleagues how to order antihemorrhagic drugs in *emergency situations* due to not knowing how to	PC	↓	↓	↓
Procedural knowledge	Requesting colleagues to review draft orders (eg, allergy tests) due to being uncertain whether the draft orders have been entered properly	PC	↑	•	↓
	Entering patient data via progress notes due to being unsure how to use certain EHR functionalities (eg, family history matrix)	PC	↓	↓	•
	Entering the same patient data in 2 near-identical data fields due to being unsure which data field entry will be forwarded to the right colleague	C	•	•	↓
	Rebooting the EHR due to not knowing how to efficiently navigate back to the main screen	C	•	•	↓
	Purposefully ordering too great a quantity of drugs (eg, 2 tubes instead of 1) due to being unsure of what quantity will eventually be delivered	P	↓	↓	↓
Memory aid	Temporarily boldfacing, italicizing, or underling parts of text in progress notes as a memory aid for questions to be asked or appointments made	CO	•	•	•
	Writing down keywords in a patient’s progress note in advance of an outpatient consultation session as a reminder	C	•	•	•
	Writing patient data from other EHR tabs or external information systems down on paper as a memory aid to avoid excessive toggling between EHR tabs or windows while writing a progress note	C	↓	•	↑
Awareness	Purposefully entering patient data perceived important for other colleagues to see in data fields that are directly shown on the user’s screen when opening a patient’s health record, rather than in the intended field(s)	PCO	↓	↓	↑
	Bookmarking scheduled patient consultation sessions with specific colors, indicating these patients will be seen by clinicians not yet having a personal identity	CO	•	•	•
	Writing specific patient data down on paper next to entering this into the EHR as a heads-up for the following clinician seeing the patient afterwards	C	•	•	↓
Social norms	Copying a workaround after having heard of or seen a workaround being used by a colleague in practice (eg, entering patient data into a data field supposed to be exclusively used by another specialty)	C	?	?	↓
	Entering patient data (eg, allergies or vital signs) into an inappropriate data field as commanded by a superior, without entering these data into the appropriate data field(s)	PCO	↓	↓	?
	Entering patient data (eg, allergies or vital signs) into an inappropriate data field as requested by a fellow clinician, in addition to entering these data into the appropriate data field(s)	PCO	?	?	↓

##### Memory Aid

To remind oneself, EHR users would apply temporary mark-up to parts of text. Specifically, physicians and nurses were observed to temporarily boldface, italicize, or underline specific parts of text in progress notes as a reminder for them to, for instance, ask specific questions or plan a follow-up appointment. This information was supposed to be removed when finalizing the progress note. However, this was sometimes forgotten, causing fellow clinicians from both within and outside of the specialty of the EHR user to think an appointment still had to be planned or specific information asked when reviewing the progress note.

##### Awareness

EHR users would purposefully enter patient data they perceived important for other colleagues to see in data fields other than the intended data field(s). For example, physicians and nurses entered important patient data in a data field that is strictly meant for listing patient discharge criteria. Data entered in this field are directly shown on the EHR user’s screen when opening a patient’s health record, making this an attractive field to store important data and draw attention. However, as soon as a patient is (re)admitted to the hospital and the important data stored into the patient discharge criteria field are replaced by actual discharge criteria by another clinician, these data are lost and no longer visible, thereby jeopardizing patient safety.

##### Social Norms

EHR users mimicked workaround behavior from their colleagues. This primarily occurred either after having heard of or seen a workaround being used by a colleague. For example, most physicians generated lists of patients with identical medical conditions (based on patient data present in the system). Within these lists, much to their frustration, physicians were unable to add additional free text alongside each patient entry in the top-level overview. Physicians argued this hampered them in efficiently searching through their patient lists, as they had to look into each patient entry one by one. A physician heard from a colleague that the neonatology group managed to add free text to each patient entry by looking into the property menu of each patient entry and selecting “NICU note” (neonatal intensive care unit)—a functionality developed by the EHR vendor as requested by the neonatology group. Free text could then be entered in a field that would be shown alongside each patient in the top-level overview. The physician managed to find this hidden functionality and shared her knowledge of this workaround with her colleagues working outside of the neonatology department. These colleagues in turn rapidly copied this workaround behavior, much to the annoyance of the neonatology staff who consider this abusive use of their data field polluting their own patient records.

**Table 4:** Prominent EHR workarounds concerning Technology and Tools and their scope and potential impact (↑ denotes an increase, ↓ denotes a decrease, • denotes a negligible influence, and ? denotes undecided. P stands for patient, C for health care professional [clinician or clerk], and O for the overall organization).

Rationale	Workaround	Scope	Safety	Effectiveness	Efficiency
Usability	Copy-pasting patient data from previous progress notes into a new progress note and subsequently modifying and supplementing these data due to usability issues with the standardized data entry template	PCO	↓	↓	•
	Manually planning (follow-up) appointments due to the automatic planning functionality providing bad visibility and oversight	C	•	↓	↓
	Postponing order entry in the EHR system during phone calls with patients as the EHR phone call interface does not accept orders	C	?	↓	↓
Technical issues	Writing down important information on paper and reentering this information into the EHR after the system crashes as booting backup takes too long	C	↓	↓	↓
	Registering batches of patient bleedings in a tailor-made standalone database as the EHR only accepts 1 bleeding registration per minute	PCO	↓	↓	•
	Either being informed by a colleague or regularly manually checking whether an expected patient had arrived in the waiting room as the arrival notification system is broken	PCO	•	•	↓
	Redrawing hemophilia family trees on paper due to failed data migration from the system used before the EHR and the current EHR	C	•	↓	↓
	Reentering orders into EHR after hardware-related printing issues, as orders are marked completed after print orders and cannot be printed again	PCO	↓	•	↓
	Repetitively adjusting predefined order sets because they contain known mistakes	PC	↓	↓	↓
Data presentation	Manually editing automatically generated letters because of, for example, undesirable font type, size, color, or order in which data are listed	CO	•	•	↓
	Drawing graphs on paper as the EHR was unable to generate the desired chart or graph (eg, line chart instead of pie chart)	PC	•	↓	↓
	Textually describing affected joints or connective tissues by rheumatology in a patient’s progress note due to absence of a virtual body	C	•	↓	↓
	Drawing a body on paper and indicating affected joints or connective tissues by rheumatology and subsequently scanning and importing this into her	C	•	↓	↓
Patient data specificity	Further specifying patient data (eg, race, allergies, and social history) in progress notes because the standardized data entry template does not facilitate a sufficient specificity level	PC	↓	↓	•
	Skipping data fields in the standardized data entry template because they are considered inapplicable or irrelevant to the patient being seen (eg, smoking or drug use history when seeing a toddler)	C	•	•	↑

#### Technology and Tools

Four EHR workaround rationales primarily relate to the Technology and Tools component. That is, these workaround rationales resonate most closely with the EHR [52]. An overview of prominent Technology- and Tools-related workarounds and their scope and impact per workaround rationale related to this component is provided in Table 4.

##### Usability

Usability-related workarounds were devised when clinicians experienced user interface-related challenges while accomplishing a task in the EHR. For example, the EHR offers an extensive standardized data entry template for medical record keeping. However, whenever patient data had to be entered into the EHR during or after each patient visit, nearly all observed users preferred copy-pasting patient data from previous progress notes into a new progress note and subsequently modifying and supplementing this data. Only a selected portion of data was entered via the standardized data entry template (eg, vaccinations, medical diagnoses, current medication, and orders) as this is required as per the hospital policy. Reasons given for not using the standardized template include excessive up and down scrolling within the template due to the order of data fields presented to the user being misaligned with workflows in practice, inconsistent and confusing placement of user interface elements (eg, sign or agree buttons), and the template containing too much irrelevant screen clutter (eg, information or functionalities deemed entirely irrelevant). Although users preferred to enter data via progress notes over using the standardized template, clinicians argued this practice causes patient data to get lost in the system over time. As commented by a physician:

For this patient, over 25 progress notes were created this week. […] We rarely copy-paste all information from an existing progress note into a newly created progress note. So, I am afraid important information simply gets lost in the EHR over time.

##### Technical Issues

Technical issues related to the EHR hindered users in accomplishing their tasks. For instance, multiple physicians occasionally experienced their EHR to crash whenever they loaded the growth analyzer functionality used to document, monitor, and analyze the growth and development of patients. Because booting the entire system backup again took minutes to complete, the physicians would write down important information on paper and reenter this information into the EHR either after the patient left the room or later that same day. Another example concerned the patient arrival notification system not automatically updating itself as it should have. As a result, clinicians had to either regularly manually check whether an expected patient had arrived in the waiting room or be informed by a colleague (eg, a clerk) that the patient had arrived.

##### Data Presentation

Data presentation workarounds relate to instances where either data in a patient’s health record were not presented to the clinician in line with expectations or when the EHR was incapable of presenting the data in a way preferred by the clinician. In both cases, clinicians would (re)organize or (re)visualize the data themselves—often on paper. For example, an infectious disease physician wanted to show a patient’s blood test results over a specific period of time by means of a graph. However, the EHR was unable to generate any charts or graphs, and as a result, the clinician herself had to draw graphs on paper.

##### Patient Data Specificity

Clinicians experienced the EHR to occasionally prevent them from being sufficiently specific when entering patient data. For instance, a physician had to specify a patient’s race in the EHR. The EHR offers a range of possible races from which 1 option can be selected. Although the available and applicable option mixed race could be chosen, the physician argued that “mixed doesn’t really tell us anything. I’d rather just write down that her father is Moroccan and that her mother is Dutch.” The physician decided to further specify the patient’s race in a newly created progress note. However, because the number of progress notes tends to increase quickly over time, these data may sooner or later be overlooked and thereby jeopardize patient safety.

#### Tasks

Task-related workarounds were driven by factors related to the tasks performed by physicians, nurses, or clerks while using the EHR. Among these factors are workload, time pressure, job content, cognitive load, and needs for attention [52]. Two workaround rationales are associated with this component: task interference and commitment to patient interaction (see Table 5).

##### Task Interference

While having to perform multiple tasks simultaneously, EHR users would write down patient data on paper to be entered in the EHR at another moment or temporarily enter patient data in an inappropriate single data field with the intention to reenter the data into the intended data fields afterwards. In the first case, a physician argued that due to the nature of her profession, she primarily examines her patients on a treatment table rather than providing consultation from behind a computer screen. Because she cannot examine patients and enter patient data into the EHR simultaneously, she wrote all necessary patient data down on paper during examinations as a memory aid, and reentered the data into the EHR as soon as patients left the examination room. She argued that this at least doubled her registration efforts, as before the EHR was implemented, filling out a paper form during the examinations sufficed. In addition, another physician was observed to knowingly enter all relevant patient data into a single inappropriate data field. Similar to the first case, she argued that she could not enter all patient data into the appropriate data fields while interacting with her patients. She would reenter all patient data from the single data field into the appropriate data fields after patients left the room.

##### Commitment to Patient Interaction

Multiple physicians and nurses argued that they have an unintentional inclination to spend relatively more time interacting with the EHR than making eye contact with patients during a patient’s visit. A physician commented that from the perspective of a patient, seeing and having eye contact with a doctor is an important psychological aspect of a patient’s visit and well-being. As a result, clinicians decided to write down keywords during a patient’s visit and enter these data into the EHR later on as they valued face-to-face interaction with patients over immediately entering patient data into the EHR. This same rationale applied to clinicians entering as much patient data or draft orders as possible into the EHR before seeing patients. Although both workarounds were perceived to be less efficient, clinicians argued this was offset by increased quality of care as they allowed them to spend more time interacting with their patients.

**Table 5:** Prominent EHR workarounds associated with Tasks and their scope and potential impact (↑ denotes an increase, ↓ denotes a decrease, • denotes a negligible influence, and ? denotes undecided. P stands for patient, C for health care professional [clinician or clerk], and O for the overall organization).

Rationale	Workaround	Scope	Safety	Effectiveness	Efficiency
Task interference	Writing patient data down on paper during examinations as a memory aid and reentering these data into the EHR after patients left the examination room, as some clinicians indicated that they cannot simultaneously examine patients and enter patient data	C	•	•	↓
	Writing patient data down on paper during telephone consultations as a memory aid and reentering these data into the EHR after the telephone conversation, as some clinicians indicated that they cannot simultaneously call and enter patient data	C	•	•	↓
	Entering all relevant patient data into a single inappropriate data field and reentering these data into the appropriate data fields after patients left the room	C	↓	•	↓
Commitment to patient interaction	Writing down keywords on paper during patient visits and entering these data into the EHR after patients left the room to spend more time interacting with patients	PC	•	↑	↓
	Entering patient data or draft orders into the EHR before seeing patients to spend more time interacting with patients	PC	•	↑	↓

**Table 6:** Prominent EHR workarounds concerning the Organization and their scope and potential impact (↑ denotes an increase, ↓ denotes a decrease, • denotes a negligible influence, and ? denotes undecided. P stands for patient, C for health care professional [clinician or clerk], and O for the overall organization).

Rationale	Workaround	Scope	Safety	Effectiveness	Efficiency
Efficiency	Not updating do not resuscitate orders as this has to be done every time a patient is readmitted to the hospital (sometimes every week)	PCO	↓	↓	↑
Data migration policy	Requesting lab results from longer than 5 years ago via an online form, as hospital management decided to not migrate lab results for more than 5 years ago to the her	PCO	↓	↓	↓
Enforced data entry	Entering patient data in progress notes rather than via the standard data entry template due to being forced to enter patient data of an unknown specificity level (eg, specific type of knee surgery a patient had 13 years ago)	PCO	↓	↓	↓
	Entering x in a mandatory data field to proceed when the supposed entry in the data field is not known or beyond one’s expertise	CO	↓	↓	↑
Required data entry option missing	Creating blank orders as multiple desired orders (eg, multivitamin supplements) are not listed in the EHR despite being available	PCO	↓	↓	↑
	Entering (a part of) a patient’s medication regimen in progress notes rather than the intended data entry fields in case the externally prescribed medication is not recognized by the EHR	PCO	↓	↓	↓
	Ordering a too low or too high drug dose enforced by technical limitations and entering a textual description in multiple data fields that the supposed dosage should be, for example, 3.75 mg per day instead of the ordered 2.5 mg per day	PCO	↓	↓	↓
	Entering a diagnosis that most closely resonates with the actual diagnosis as the desired data entry option is not offered	PCO	↓	↓	•
	Writing allergy-related patient information down in a progress note as the required allergy is not in the list of to-be-chosen allergies	PCO	↓	↓	•
	Leaving data field blank when the right option for “Reason for stopping medication” is not there in the drop-down list when stopping medication	PCO	↓	?	•

#### Organization

Workaround rationales associated with the Organization component of the work system stem from the organizational conditions in which EHR usage occurs [52]. Examples of organizational factors unintendedly driving the creation of workarounds are organizational and patient safety culture, supervisory and management style, and rules and regulations. Four EHR workaround rationales are associated with this component: efficiency, data migration policy, enforced data entry, and required data entry option missing (see Table 6).

##### Efficiency

Clinicians created workarounds to improve their actual efficiency of accomplishing tasks with the EHR. For example, clinicians knowingly did not reenter do not resuscitate (DNR) orders in the EHR. Although DNR orders are valid for up to 1 year, the EHR requires clinicians—as a result of the hospital policy—to reenter DNR orders every time a patient is readmitted to the hospital. In several cases, patients were readmitted every week on a routine basis. However, clinicians considered reentering DNR orders for such patients on a weekly basis a “waste of time” and therefore only entered a DNR order once. This order was only reentered upon request by the patient or after the order became invalid after a year. Although this practice made workflows of clinicians at hand more efficient, patient safety and effectiveness of care diminish as patients may change their mind about their DNR order after a week without explicitly communicating this to their clinician(s) (the latter being the main reason why this DNR reentry policy is enforced).

##### Data Migration Policy

Multiple clinicians felt enforced to request (essential) historical data because of data migration policy decisions taken by the hospital management team during the design and implementation phase of the EHR. For example, only lab results dating back to a maximum of 5 years were to be migrated to the new EHR. Multiple hematologists argued that “In order to determine the right dosage for our hemophilia patients, it is paramount that we know the antibody values of our patients against specific drug types, basically from their moment of birth till the present.” To gain access to lab results entered into the system used before the current EHR for more than 5 years ago, clinicians have to fill out an online form that takes 5-10 minutes of their time. The processing of these forms is estimated to require additional 3 days. Not only does this negatively impact efficiency, but it also poses direct threats to patient safety in emergency situations where the right dosage of a drug cannot be accurately determined due to the absence of historical lab results data.

##### Enforced Data Entry

Clinicians occasionally experienced the EHR to force them to be overly specific when entering patient data. For instance, a patient told a physician to have had knee surgery back in 2003. When entering this information into the EHR, the physician was forced to specify the precise type of knee surgery from a multitude of possible options. Both the physician and patient were unsure of the exact type of knee surgery and the physician was unable to simply enter ‘knee surgery’. This required specificity level of data entry did not stem from technical limitations, but was enforced by the hospital policy as the options for knee surgery from which the physician can choose are linked to the types of knee surgeries performed within the hospital. The physician decided to enter these data in a progress note rather than in the appropriate data field.

##### Required Data Entry Option Missing

The EHR occasionally did not offer data entry options desired by clinicians, particularly when ordering medication, altering a patient’s current medication regimen, or entering symptoms into the patient’s Problem List. For example, a physician wanted to order 3.75 mg of prednisone (1.5 tablets) per day for a patient. However, the EHR did not accept 3.75 mg and forced the physician to choose from either 2.5 mg (1 tablet) or 5 mg (2 tablets) per day. According to the physician, the EHR does not understand that the 2.5 mg tablets can be easily broken into half by patients. As the desired option of 3.75 mg was unavailable, the physician ordered 2.5 mg per day but entered a textual description in multiple data fields that the supposed dosage should be 3.75 mg per day. Although this workaround solved the workflow mismatch at the time, the physician commented to be “one hundred percent sure” that a medication error will occur to one of his patients sooner or later. “If one of my patients would be (re)admitted to hospital and the attending physician would only notice the EHR-enforced prescribed dosage of 2.5 mg of prednisone per day in the patient’s medication overview rather than the prescribed dosage of 3.75 mg per day in the textual description, you can imagine what kinds of mistakes could be made.” Upon closer inspection, it turned out that the root cause of this workaround did not result from the fact that the EHR could not process the physician’s order. Instead, the drug ordering functionality of EHR is purposefully programmed this way as a result of the hospital policy as the list of all possible drugs to be ordered are derived from the inventory of the hospital pharmacy.

#### Physical Environment

The Physical Environment component of the work system refers to the environment and its conditions in which various tasks are carried out [52]. We observed and interviewed the case study participants in 3 distinct physical environments: private offices, examination rooms, and inpatient wards. However, no workaround rationales were associated with these physical environments or their conditions such as room layout, noise, lighting, temperature, or work station design.

## Discussion

### Contribution

Health care providers resort to informal work practices known as workarounds to handle exceptions to normal workflow unintendedly imposed by EHRs. Although workarounds may occasionally be favorable [27,30,38], they are generally suboptimal and may jeopardize patient safety, effectiveness of care, and efficiency of care [31-33,39-41]. Given their potentially adverse impact, understanding why and how workarounds occur is pivotal to develop user-friendly EHRs and achieve greater alignment between work contexts and EHRs.

Existing literature primarily provides insight into multiple reasons behind EHR workaround creation [21,36,48-51] and describes several key features of EHR workarounds [47]. However, research into the scope and impact of EHR workarounds on patient care processes is not as extensive. This narrows our understanding of the effects EHR workarounds have on the organizational workflows and outcomes of care services. This study contributes to the body of literature on EHR workarounds in 2 ways. First, we presented 15 bottom-up identified rationales for EHR workarounds. Our bottom-up approach meant looking at data in an open-minded way that led to the identification of 3 rationales that hitherto had not been identified by prior studies. Second, for each workaround rationale, we analyzed workarounds on their scope and impact from a sociotechnical perspective using SEIPS as a reference framework [52].

### Identified Rationales for Electronic Health Record System Workarounds

After coding our data using a bottom-up approach, we compared our results with those in existing literature on EHR workaround rationales to look for commonalities as well as differences. Concerning similarities, multiple of our rationales have also been described with identical terms in prior studies which have identified workarounds related to memory aid [21,36,37,48,65], awareness [21,36,37,48], efficiency [21,36,37,48], patient data specificity [21,36,48], commitment to patient interaction (termed “sensorimotor preferences”) [36,48], required data entry options missing (termed “no correct path”) [21], technical issues [51], and social norms (termed “cultural factors”) [51]. In addition, in prior studies, our EHR workaround rationales “declarative knowledge,” “procedural knowledge,” and “usability” have been separately categorized [51] as well as merged in a single rationale [21,36,37,48]: “knowledge/skill/ease of use.” On the basis of our dataset, we found that “declarative knowledge,” “procedural knowledge,” and “usability” are rationales for workarounds that can be clearly distinguished and demand to be tackled in a different way.

Our workaround rationale taxonomy may be more refined compared with those in existing literature. Despite identical naming, existing examples of the rationale “efficiency” [21,36,37,48] may not be identical or applicable to our rationale “efficiency.” For example, Flanagan ME et al [21] mention that their most frequently encountered example of computer-based efficiency workarounds concerned users “copying and pasting text from previous progress notes into a new progress note.” Although our observed clinicians did the exact same thing, we found that the rationale for this workaround was actually “usability” because the clinicians favored entering and copy-pasting the majority of patient data in progress notes due to low perceived usability of the standardized data entry template. In our study, only workarounds to which no other underlying rationales were applicable but to purely accomplish a task with greater efficiency were labeled as “efficiency.”

Our rationales “data migration policy,” “enforced data entry,” and “task interference” do not directly correspond with rationales identified in existing literature. In contrast, our dataset provided no evidence of workarounds that could be directly related to task complexity [21,36,48,51], longitudinal data processes [21,36,48], trust [21,36], security [36,51], EHR vendor contract-related issues [51], or double or duplicate documentation due to hospitals using multiple incompatible EHRs [50]. However, not all workaround rationales identified in prior research apply to our hospital setting. For example, we did not identify the workaround rationales “trust” (defined as “greater trust in paper over electronic version”) [21,36,37] or “security” (defined as “security associated with the EHR encourages paper use as an alternative”) [36,51] because any paper-based orders are no longer and in no way accepted in the case study hospital. EHR users therefore have no other option but to create computer-based rather than paper-based workarounds to proceed with their workflow when placing orders. In addition, we found no workaround rationales that could be associated with the Physical Environment component of the SEIPS framework. This could be due to the nature of the EHR studied, contrary to, for example, studies investigating workarounds to barcode medication administration (BCMA) systems. For example, physical factors to BCMA workarounds such as unreadable medication barcodes (eg, crinkled, missing, and torn), unreadable or missing patient identification wristbands (eg, chewed, soaked, and self-removed), or loud ambient noise preventing nurses from hearing scanner alarms [33,66] were not applicable in any of the 3 physical environments in which we observed EHR usage.

### Scope and Impact of EHR Workarounds

Three interesting observations can be made regarding the scope and potential impact of workarounds on patient safety, effectiveness of care, and efficiency of care. First, nearly all observed workarounds except for those related to the rationale “social norms” could have a positive or negative impact on at least one of these 3 dimensions. The potential impact of workarounds should therefore not be underestimated. Second, all workarounds related to the rationales “enforced data entry” and “required data entry option missing” could reduce patient safety. Likewise, all workarounds related to the rationales “enforced data entry,” “required data entry option missing,” “usability,” and “data presentation” could reduce the effectiveness of care. All workarounds related to the rationales “task interference,” “commitment to patient interaction,” “technical issues,” and “data presentation” could reduce the efficiency of care. Third, tradeoffs could also be seen between the 3 dimensions. For example, all workarounds related to the rationale “commitment to patient interaction” showed an increase in effectiveness of care at the expense of efficiency of care. Workarounds should therefore be assessed with care from multiple perspectives.

In summary, knowing the scope as well as impact of each workaround aids health care practitioners and other stakeholders such as EHR developers or management in prioritizing the handling of workarounds. For example, in our case study hospital, multidisciplinary teams consisting of among others physicians, nurses, quality assurance staff, and EHR developers work together to identify, analyze, and resolve workarounds. A well-defined workflow in a specific specialty such as medication ordering in the gynecology department is generally taken as a starting point for workaround identification and analysis. If the perceived potential improvements of resolving the workarounds are deemed satisfactory, the identified workarounds are studied more broadly in other specialties as well to see if hospital-wide improvements could be achieved. Workarounds that are found to affect patients or have the potential to negatively impact patient safety are resolved first as patient safety is concern number one. Likewise, workarounds that affect patients and have the potential to improve patient safety are sustained and, if possible, integrated in user-EHR workflows. It should be taken into account that our definitions of patient safety, effectiveness of care, and efficiency of care may not be directly applicable to other hospitals. Results should therefore be interpreted with care.

### Added Value of the SEIPS Framework

Concerning the sociotechnical perspective, we used an adapted version of the SEIPS framework [52] tailored to our context to interpret, analyze, and determine the scope and impact of each EHR workaround. The integrative and holistic perspective of the SEIPS framework proved useful to study workarounds in relation to not just the health care work system in which they were created but also in relation to the care processes performed and resulting outcomes on patient safety, effectiveness of care, and efficiency of care. This was beneficial for 3 main reasons.

First, the SEIPS framework allowed us to indicate what workaround rationales are most closely associated with each of the 5 components of the work system. This aided us in more accurately determining how each workaround could be resolved. For example, workaround rationales associated with the Persons component include a person’s declarative knowledge and procedural knowledge of using the EHR. Such workarounds may most effectively be resolved through personal training to assure optimal and proper EHR use. Likewise, workaround rationales associated with the Organization component may most effectively be resolved through reviewing organizational policy and regulations and subsequently EHR data entry policies, whereas Tasks-related workarounds may most effectively be resolved through task redesign. Workaround rationales associated with the Technologies and Tools component were primarily the result of clinicians bringing their own workflow in line with the EHR-dictated workflow, as the latter is relatively fixed. These workarounds may, therefore, most effectively be resolved through EHR redesign efforts. However, it should be taken into account that workarounds must be thoroughly assessed before they are classified under 1 of the 5 work systems components of the SEIPS framework. For example, “required data entry option missing” workarounds seemed to be related to Technology and Tools-related workarounds at first sight. Upon closer inspection, however, it turned out that the root cause of these workarounds did not result from the EHR not being able to, for example, process physicians’ orders or list additional data entry options. Instead, the drug ordering functionality of the EHR is purposefully programmed this way as a result of the hospital policy as the list of all possible drugs to be ordered are derived from the inventory of the hospital pharmacy—making them Organization-related workarounds.

Second and related to the foregoing, the SEIPS framework is supportive in planning these redesign efforts of the work system. Multidisciplinary teams of physicians, nurses, quality assurance staff, and EHR developers, as aforementioned, reflect on the current configuration of the work system to prevent unfavorable workarounds from occurring. Likewise, future work system configurations are discussed to, for example, explore how a redesign of the EHR would impact interactions between the work system’s people, tasks, organization, other tools and technologies, and internal and external environmental factors.

Finally, the adapted SEIPS framework including the workaround rationales associated with each work system component in Figure 2 is a snapshot of the studied sociotechnical system based on approximately 14 months of observations and interviews. Because EHR workarounds are subject to gradual change caused by, for example, changes in one’s knowledge of the EHR, personal preferences, regulations, policy, care directives, or financing structures of the hospital, workarounds are not set in stone and may change over time. Multiple snapshots are being taken over time and compared in search of interesting clues about the evolution of workarounds and implications hereof in practice.

### Limitations of the Study

This study has several limitations. First, this study was performed at a large academic hospital. EHRs in academic hospitals tend to be more complex than their nonacademic counterparts, as they must cater to the needs of many diverse highly specialized patient care practices each with varying electronic functionalities [67]. Larger hospitals also tend to have access to more sophisticated and tailor-made EHRs including a large pool of technology-support personnel, contrary to smaller care practices generally relying on commercially available EHRs with less functionalities and limited information technology sources [47]. This means the results should be interpreted with care and may not be applicable to other health care contexts.

Second, the EHR studied had been in use for around half a year from the moment our first observations began. Although the case study participants indicated to be largely past the valley of despair [68], workaround rationales became increasingly or decreasingly prevalent as time progressed. For example, workarounds created due to a lack of declarative or procedural knowledge of using the EHR occurred far more frequently than the other types of workarounds within the first months of observation. These workarounds became less prevalent as case study participants steadily became more proficient in using the EHR while our observations continued for over a year. The greater the user proficiency with the EHR, the more other rationales for workarounds such as the need to enter patient data with greater or lesser specificity or preferring alternative ways of data presentation came to the fore.

Third, we may not have captured all workarounds used in practice. However, the observations and interviews continued till the research team (VB, KK, MW, and MJ) agreed that data saturation was achieved. This is confirmed by the large number and broad variety of workarounds we observed. This led to the development of a solid set of workaround rationales that can be used to analyze workarounds that we may not have seen during our observations or interviews.

Finally, to mitigate the Hawthorne effect during observations and audiovisual recordings, we clearly communicated to the participants what is in it for them. We explained how participating in the research project was an opportunity to improve the EHR and thereby reduce potentially negative impacts on patient safety, effectiveness of care, and efficiency of care. We also stressed that we were observing the EHR rather than the participant and clearly communicated that data gathered are made fully anonymous, cannot be traced back to them, and will not be shared with anyone else not belonging to the research team. This reassured the participants to use their EHR as they normally would without fear of potentially being reprimanded or rebuked after participation. Participants actually commented to be glad that research was being performed on EHR workarounds because they were aware of their potentially hazardous effects. Finally, the audiovisual camera was permanently and unobtrusively installed for the duration of the observations and interviews, and did not require frequent maintenance or recalibration. Observers were positioned at a safe distance from the clinician using the EHR (see [69]).

### Future Research

Further research is currently being performed for the identification of key features of the identified EHR workarounds. Examples of such features include their cascadedness (ie, whether a workaround is stand-alone or initiates a series of additional workarounds), avoidability, anticipatedness, and repetitiveness. This knowledge may then be used to better understand the implications EHR workarounds may have on the safe, effective, and efficient delivery of care to patients, as well as aid in subsequently determining how they should be handled.

Two main recommendations for future research can be given. First, additional observational studies using a top-down approach (eg, [21,37,48] for top-down approaches) could be performed to see whether the coding taxonomy containing the 15 EHR workaround rationales could be refined or extended. Second, future research could also study how changes in work system component-related factors such as EHR user training, physical workspace layout, organizational policies, task content, or redesign efforts of the EHR could result in a more balanced and close fit between the various work system components.

## Abbreviations

BCMA: barcode medication administration
DNR: do not resuscitate
EHR: electronic health record system
NICU: neonatal intensive care unit
SEIPS: Systems Engineering Initiative for Patient Safety
